# Dairy Consumption Across Generations—A Structural Equation Model of Central European Adults

**DOI:** 10.3390/nu18193226

**Published:** 2026-09-30

**Authors:** Ewa Halicka, Małgorzata Kosicka-Gębska, Jerzy Gębski, Krystyna Rejman

**Affiliations:** Institute of Human Nutrition Sciences, Warsaw University of Life Sciences WULS-SGGW, Nowoursynowska 159c, 02-776 Warsaw, Poland

**Keywords:** dairy consumption, Baby Boomers, Generation X, Millennials, Gen Z, nutritional value, health, consumer innovativeness, sustainability, Structural Equation Modeling (SEM)

## Abstract

**Background/Objectives:** Dairy products provide a wide range of essential nutrients and therefore remain relevant to nutrient adequacy and public health challenges. Concurrently, consumer behaviors vary across demographics and continue to evolve in response to the transformation of the food system as a whole. **Aim and Methods:** To examine the impact of health and sustainability, as well as consumers’ openness to innovations and food choices from a generational perspective, data collected among 3131 adults were analyzed. The link between dairy consumption and three latent variables was evaluated using Structural Equation Modeling (SEM). **Results:** The clearest pattern was observed among Gen X, where all three constructs—nutritional value, innovativeness, and sustainability—were positive predictors of dairy consumption. Sustainability was a strong, significant predictor among Millennials, and none of the analyzed three variables was statistically significant among the Baby Boomers and Gen Z cohorts. **Conclusions**: The study demonstrates that dairy consumption among Central European consumers is a multidimensional phenomenon impacted by multiple determinants. The patterns of associations observed across generational and gender subsamples were not uniform, highlighting the importance of considering consumer diversity when developing communication tools and public health nutrition policies.

## 1. Introduction

Dairy products, including milk, are a recognized part of the human diet and one of the main sources of high-quality protein, calcium, phosphorus, potassium, and vitamins [1]. They are also a source of bioactive peptides, prebiotics, and beneficial bacteria and can help prevent several diseases, including osteoporosis. The milk fat globule membrane (MFGM), a component not present in other foods, plays a key role in lipid metabolism and neuronal development. Regular consumption of this nutrient-rich group of foods may play a significant role in maintaining bone health, preserving muscle mass, and meeting nutritional requirements across all ages. Milk intake is also associated with a lower risk of cardiovascular disease, stroke, hypertension, colorectal cancer, metabolic syndrome, obesity, and osteoporosis [2].

Despite the significant nutritional value of traditional dairy products, a shift towards plant-based alternatives has been observed in many highly developed countries in recent years [3]. Based on FAO data, the yearly per capita supply of milk products, excluding butter, in the European Union has continued to increase over the last 15 years, from 197 kg in 2010 to 232 kg in 2024 [4]. The consumption of fermented dairy products, products with enhanced functional properties, and plant-based milk alternatives is also rising [5]. These changes are a consequence of social transformations, growing health awareness among consumers, lifestyle changes, and evolving dietary preferences [3,6,7].

A growing body of research suggests that eating habits differ between generations, which constitute an important demographic variable at the population level. Deep generational divergence was documented in another study conducted in 2024, which concluded that food suppliers should adopt a targeted, selective approach to behavioral profiles [8]. In this context, it is also important to understand the influence that older generations have on shaping the behaviors of those younger than them. Parents tend to raise their kids with the same belief systems as their own—beliefs that are based on the parents’ worldview and serve as a factor in the child’s development of their own worldview [9]. Each generation has grown up in different social, economic, and cultural circumstances, which have shaped their eating habits, their perceptions of food, and the factors influencing their purchasing decisions. Older generations are more likely to be guided by habit, tradition, and the perceived nutritional value of products, while younger consumers pay greater attention to health and environmental considerations, product innovation, and convenience [10]. If Generation Z can be described as “mobile natives,” Generation Alpha (born from 2010 onwards) might be more aptly defined as “algorithmic natives” or even “AI natives,” given that their earliest technological experiences are increasingly shaped by intelligent assistants, pre-curated content streams, and ambient computing [11]. Generation Z is exposed to sustainability challenges (including environmental, social, and economic) and to negative health outcomes from food choices, like no other generation [12]. Changes in dairy product consumption result from the interplay of multiple concurrent factors [10,13]. The most frequently cited include health status, lactose intolerance, milk protein allergy, high nutritional value of dairy products, economic situation, the presence of children in the household, and consumer preferences. Individual psychographic characteristics, such as openness to food innovation and concern for the environment, are also becoming increasingly important [14]. Consumers with higher levels of innovativeness are more likely to choose new dairy products and functional foods [15], while those more aware of the environmental impact of food production are more likely to take environmental considerations into account when making purchasing decisions. However, these factors may play different roles across generations.

Exploring factors that influence dairy product consumption across different age groups is of great importance from both public health and dairy sector perspectives [13,14]. A better understanding of what drives dairy product choices across generations can facilitate the development of more effective dietary guidelines, the design of age-tailored educational initiatives, and the creation of products that meet the expectations of specific consumer groups. This knowledge can also help food producers and policymakers responsible for nutrition policy to develop measures tailored to the demographic changes taking place in Europe. Poland, Germany, and the Czech Republic constitute a particularly interesting area for research into consumer behavior. These countries operate within similar legal and economic frameworks resulting from their membership in the European Union. All three are also experiencing significant demographic changes and generational shifts among consumers, which influence the development of the region’s dairy market.

Despite the growing number of studies on dairy product consumption, there remains a lack of analyses that simultaneously consider health and nutritional behavioral motives, consumer innovativeness, environmental concerns, and their interrelationships with dairy product consumption across different generational groups [16,17]. Previous studies have most often focused on individual determinants of consumer behavior or have been conducted in a single country [13,18]. Little is known about whether the mechanisms that determine dairy product consumption are similar across generations. Generational affiliation may be one of the most significant factors differentiating contemporary patterns of dairy product consumption.

To the best of the authors’ knowledge, this is one of the first studies to compare the relationships between motives for choosing dairy products and consumption patterns across four generations living in Central Europe.

## 2. Materials and Methods

### 2.1. Data Collection Process

The study was conducted in December 2024 using an online questionnaire administered via Computer-Assisted Web Interviewing (CAWI). Data were collected simultaneously in Poland, Germany, and the Czech Republic. These countries were selected because they represent European Union (EU) Member States with comparable historical, socio-economic, and cultural backgrounds. Currently, Poland is one of the largest milk producers in the European Union [7,19], while Germany is among the leading European markets for plant-based alternatives. The Czech Republic shares similar dairy consumption traditions with Poland, but exhibits distinct consumer trends, including higher per capita milk consumption and different patterns of acceptance of plant-based dairy alternatives [20]. The growing trend towards plant-based diets is increasingly evident in the Czech Republic as well. According to national surveys, in 2022, 3% of Czech consumers identified themselves as vegan, 7% as vegetarian, and 25% as flexitarian [21].

A total of 3131 adults participated in the study, including 1508 respondents from Poland (48.2%), 812 from Germany (25.9%), and 811 from the Czech Republic (25.9%). Data collection was carried out by a professional market research agency, ARC Rynek i Opinia, using its proprietary online research panel (ePanel.pl), comprising approximately 50,000 registered users. Panel members are recruited through both online and offline channels, and the panel’s average response rate is approximately 20% [22].

Quota sampling was used to obtain samples that reflect the demographic structure of each country. Quotas were established for gender, age, education level, and place of residence based on official national statistics. For Poland, demographic benchmarks were derived from the 2021 National Population and Housing Census. Corresponding data for Germany and the Czech Republic were obtained from the Federal Statistical Office of Germany and the Czech Statistical Office, respectively [23,24].

As part of the screening procedure, respondents were required to confirm that they were fully or partially responsible for food purchasing decisions within their households. Individuals not involved in food shopping decisions were excluded from participating in the study. The gender, age, and generation of the respondents are presented in Table 1.

### 2.2. Ethical Approval

The study protocol was approved on 5 July 2024 by the Rector’s Committee for the Ethics of Research Involving Human Participants at Warsaw University of Life Sciences (approval no. 31/RKE/2024). Written informed consent was obtained from all participants by the market research agency prior to participation.

### 2.3. Description of Questionnaire

The questionnaire was originally developed in Polish and subsequently translated into German and Czech for administration in Poland, Germany, and the Czech Republic. It comprised questions assessing the frequency of milk and fermented dairy product consumption, changes in dairy consumption, perceived nutritional value, consumer innovativeness, and sustainability-related considerations in food choices.

Only individuals responsible for household food purchasing decisions were eligible to participate in the study. Respondents who declared that food purchases were made exclusively by other household members were excluded from further participation.

To assess the frequency of consumption of liquid milk and fermented dairy beverages, respondents were asked to select one of six alternatives: never; 1–3 times per month or more often; once a week; several times a week; once a day; or several times a day. They also evaluated the changes in their dairy (including fluid milk) product consumption over the previous 2–3 years. The answers to these questions were used to construct the endogenous variable representing “Dairy Consumption” of the Structural Evaluation Model (Table 2).

The respondents’ motives for food choice related to the nutritional value of dairy products were assessed using 8 items selected from the Food Choice Questionnaire (FCQ) [25]. Respondents indicated the importance of the following product characteristics: beneficial to health, nutritious, rich in vitamins and minerals, high in protein, low in fat, contains no artificial additives, contains no genetically modified ingredients, and taste. The items were assessed on a seven-point Likert scale ranging from 1 (strongly disagree) to 7 (strongly agree). Statements derived from this scale were used to create the exogenous variable “Nutritional Value” in the SEM.

Consumer innovativeness was initially measured using six items selected from the Domain Specific Innovativeness Scale (DSI) [26]: “In general, I am among the last people in my circle of friends to purchase a new dairy product (R)”; “If I heard that a new dairy product was available in a local store, I would be interested in buying it”; “Compared with my friends, I am less likely to buy new dairy products (R)”; “I would consider buying a new dairy product even if I had not heard of it before”; “In general, I am among the last people in my circle of friends to know the names or brands of new dairy products (R)”; and “I know more about new products than others do.” The three negatively worded items were reverse-coded, while the remaining three items were retained in their original direction, so that higher scores consistently indicated greater consumer innovativeness toward dairy products. Following the confirmatory factor analysis, three items with inadequate factor loadings were removed. Consequently, the Innovativeness construct in the final SEM was measured using three items. Responses were rated on a five-point Likert scale ranging from 1 (strongly disagree) to 5 (strongly agree).

Sustainability-related food choice factors were assessed using six items adapted from the Sustainable Food Choice Questionnaire (SUS-FCQ) [27]. The surveyed consumers—also in this case—indicated on a five-point Likert scale (from 1—I strongly disagree to 5—I strongly agree) their reactions to 6 statements: “I choose dairy products that are produced in an environmentally friendly way”; “I pay attention to whether dairy products are packaged in an environmentally friendly way”; “I consider the place of origin of dairy products to reduce transport distances and their carbon footprint”; “I choose dairy products with quality certifications”; “Environmental pollution affects my daily life”; and “I am aware of the impact of food consumption on planetary health”. These were used to create the third SEM exogenous variable, “Sustainability.”

To classify respondents into generational cohorts, they were asked to report their year of birth. These included: Baby Boomers (born between 1946 and 1964), Generation X (1965–1980), Generation Y (Millennials) (1981–1996), and Generation Z (1997–2006).

The questionnaire was administered using the CADAS survey platform in the respondents’ native languages. To ensure high data quality, several quality control procedures were implemented, including mandatory responses to all questionnaire items, automated checks for logical consistency, and identification of contradictory or inconsistent responses.

### 2.4. Statistical Analysis

Descriptive statistics were used to characterize the study sample, and differences in categorical variables were assessed using the chi-square test of independence. The measurement model was evaluated using confirmatory factor analysis (CFA). Internal consistency and convergent validity of the latent constructs were assessed using Cronbach’s alpha, composite reliability (CR), average variance extracted (AVE), and standardized factor loadings. Model fit was evaluated using χ^2^/df, CFI, TLI, RMSEA with its 90% confidence interval, and other fit indices available in AMOS. Measurement invariance across generational groups was examined using multi-group CFA by comparing the configural and metric invariance models. Metric invariance was evaluated primarily on the basis of changes in CFI and RMSEA between nested models. After establishing measurement invariance, multi-group structural equation modeling was used to compare structural relationships across generations. SEM analyses were performed in IBM SPSS v.AMOS 31 using maximum likelihood estimation. Because the data showed departures from multivariate normality, bootstrap procedures were additionally used to assess the robustness of parameter estimates.

## 3. Results

### 3.1. Characteristics of the Study Sample

The study included 3131 respondents from Poland (n = 1508), Germany (n = 812), and Czechia (n = 811). Women constituted 51.84% of the sample, and the gender distribution did not differ significantly between countries (*p* = 0.3833).

Participants who qualified as Generation Y constituted the largest generational group (35.04%), followed by Generation X (31.11%), Baby Boomers (16.95%), and Generation Z (16.90%). The median age of respondents in the generation samples was 64, 51,36 and 24 years, respectively. The generational structure differed significantly across countries (*p* < 0.0001) (Table 1).

### 3.2. Essential Validation of the Measurement Model

The constructs were assessed for internal consistency using Cronbach’s alpha. Confirmatory factor analysis (CFA) was subsequently performed to determine standardized factor loadings, composite reliability (CR), average variance extracted (AVE), convergent and discriminant validity, correlations between the constructs, and measurement model fit indices.

As some of the results were unsatisfactory—factor loadings < 0.4, CR < 0.7 and AVE < 0.5—the variables with the lowest factor loadings were removed in turn. Ultimately, to meet the above criteria, the following variables were removed from the respective constructs:

Nutritional Value—‘low in fat’;

Innovativeness—“If I heard that a new dairy product was available in a local shop, I would be interested in buying it”, “I would consider buying a new dairy product even if I had not heard of it before”, “I know more about new products than others do”;

Sustainability—“I am aware of the impact of food consumption on planetary health”.

Following the removal of the above-mentioned variables, the model’s internal fit was assessed, and the fit statistics remained within acceptable ranges: CFI = 0.917, TLI = 0.904, RMSE = 0.023. The only deviation is χ^2^/df = 6.11, the value of which is higher than the maximum reference value of 5. It should be noted, however, that this is likely due to the large sample size (n = 3131).

In the next step, to facilitate comparison, the parameters of the individual models representing the distinct generations and genders were collated. In all group models, the CFI ranged from 0.953 to 0.957, the TLI from 0.944 to 0.948, the RMSEA from 0.019 to 0.020, and the χ^2^/df from 8.81 to 4.22.

Configurational and metric invariance were also tested (Table 3).

The CFI changed only from 0.956 to 0.954 (ΔCFI = −0.002), while the RMSEA remained virtually unchanged (0.020 vs. 0.019). Despite a significant χ^2^ test of differences, the very small change in the incremental fit indices supports metric invariance across the four generational groups. A similar situation was observed for gender.

### 3.3. Results of Structural Equation Modeling

Ultimately, after removing variables with lower factor loadings from the constructs, Cronbach’s alpha values were as follows: 0.866 for Nutritional Value, 0.736 for Innovativeness and 0.833 for Sustainability.

The predictors of the endogenous variable in the SEM model (“Dairy Consumption”) included three constructs forming latent variables, namely “Nutritional Value”, “Innovativeness”, and “Sustainability” (Table 4). These indicators were selected to capture complementary dimensions of dairy-related dietary behaviors, as frequency reflects habitual consumption in general, while changes capture intake over time.

In the model developed for the entire sample (n = 3131), all the predictors analyzed were statistically significant. The latent variable “Sustainability, “with a standardized value of β = 0.237, *p* = 0.007, had the greatest positive influence on the endogenous variable “Dairy Consumption”. A significantly weaker, but also statistically significant, positive effect was observed for the predictor “Nutritional Value”, with a value of β = 0.089, *p* < 0.001. The variable “Innovativeness” had a negative effect on the dependent variable, with β = 0.092, *p* = 0.007 (Table 4).

The clearest pattern can be observed for Generation X. In this group, all three factors were positively and significantly associated with dairy consumption. “Nutritional Value” had a standardized effect of β = 0.189, “Sustainability” β = 0.161, and “Innovativeness” β = 0.209. This indicates that all three examined factors are relevant for Generation X, with “Innovativeness” showing the relatively strongest effect.

For Generation Y, the pattern was markedly different. “Sustainability” clearly emerged as the dominant factor (β = 0.344, *p* < 0.001), representing the strongest standardized effect across all generational groups. “Nutritional Value” was not significant (β = 0.047, *p* = 0.360), while “Innovativeness” showed a negative, although non-significant, coefficient (β = −0.072, *p* = 0.193). Thus, in this group, dairy consumption appears to be primarily associated with the importance attributed to sustainability.

For Generation Z, none of the three effects reached the conventional level of statistical significance (*p* < 0.05). “Nutritional Value” nevertheless showed a relatively large positive coefficient (β = 0.216, *p* = 0.081), which may be interpreted as a tendency rather than a statistically confirmed effect. “Sustainability” (β = 0.007) and “Innovativeness” (β = 0.011) showed virtually no association with dairy consumption.

Among Baby Boomers, none of the effects was statistically significant at the conventional 0.05 level. The strongest effect was observed for “Innovativeness” (β = 0.255, *p* = 0.056), followed by “Sustainability” (β = 0.229, *p* = 0.067). Both effects were close to the 0.05 significance threshold but formally remained non-significant. “Nutritional Value” showed a weaker and clearly non-significant effect (β = 0.160, *p* = 0.129).

Several differences in the observed effects should be noted. Sustainability was clearly strongest in Generation Y (β = 0.344), whereas it was virtually absent in Generation Z (β = 0.007). “Innovativeness” was positive and significant in Generation X (β = 0.209), whereas it was negative in Generation Y (β = −0.072) and nearly zero in Generation Z. In turn, “Nutritional Value” was statistically significant only in Generation X, although the numerically largest coefficient was observed in Generation Z (β = 0.216), where it did not reach statistical significance due to a greater level of uncertainty (Table 4).

Additional Structural Equation Modeling across gender also revealed differences in the statistical significance of the predictors of the “Dairy Consumption” variable.

The statistically significant predictors of dairy consumption among women (n = 1623) were “Sustainability” and “Nutritional Value”. Both variables had a positive effect on dairy consumption. A stronger association was observed for “Sustainability”, for which the standardized path coefficient was β = 0.215, *p* < 0.001. “Nutritional Value” showed a weaker but still positive and statistically significant effect, with a standardized coefficient β = 0.105, *p* = 0.025. In comparison, “Sustainability” and “Innovativeness” were statistically significant predictors of dairy consumption among men. “Sustainability” had a positive effect on milk consumption, with a standardized coefficient (β) of 0.269, *p* < 0.001. “Innovativeness” was positively associated with dairy consumption, with a standardized coefficient of β = 0.150, *p* = 0.003 (Table 5).

Constraining the effect of nutritional value on dairy consumption to equality across generations did not significantly worsen model fit, Δχ^2^(3) = 4.53, *p* = 0.209, indicating no significant generational differences in this relationship. Similarly, the equality constraint imposed on the sustainability–dairy consumption relationship did not result in a significant deterioration in model fit, Δχ^2^(3) = 6.00, *p* = 0.111.

In contrast, constraining the effect of innovativeness on dairy consumption to equality across generations resulted in a significant deterioration in model fit, Δχ^2^(3) = 28.08, *p* < 0.001. This finding indicates that the relationship between innovativeness and dairy consumption differs significantly across generational cohorts. Follow-up pairwise comparisons were therefore conducted to identify which generational groups differed significantly from one another.

No significant gender differences were found for the relationship between nutritional value and dairy consumption, Δχ^2^(1) = 0.18, *p* = 0.671. Similarly, constraining the sustainability–dairy consumption path to equality across gender groups did not significantly worsen the model fit, Δχ^2^(1) = 0.29, *p* = 0.589.

The difference in the innovativeness–dairy consumption relationship was also not statistically significant, Δχ^2^(1) = 2.98, *p* = 0.084, although the result suggested a marginal tendency toward gender-related differences.

Overall, the findings provide no evidence that gender significantly moderates the relationships between nutritional value, sustainability, innovativeness, and dairy consumption.

### 3.4. SEM Model Fit Statistics

An analysis of our results indicated an acceptable fit between the model and the empirical data and, for certain parameters, a very good fit.

The root mean square error of approximation (RMSEA) was 0.018, and its 90% confidence interval was very narrow, ranging from 0.018 to 0.019. Such a low RMSEA value indicates a very good model fit. Furthermore, the PCLOSE = 1.000 (Table 5).

It was observed that the CMIN/DF value of 4.105 exceeds the reference value of 3; however, it is lower than the less restrictive threshold of 5. The relatively high value of this ratio may be partly attributable to the large sample size (n = 3131).

The Incremental Fit Indices also confirm that the model fits well, as the CFI = 0.946, the IFI = 0.946, the Tucker–Lewis Index (TLI = 0.936) and the Normed Fit Index (NFI = 0.930), all exceed the cut-off value of 0.9 (Table 6).

Taking the above indicators into account, it can be concluded that the model fits the empirical data well.

## 4. Discussion

The SEM results indicated that all three constructs were significant positive predictors of dairy consumption in the total sample. The generation-specific models showed different within-group patterns of association. However, formal cross-group comparisons demonstrated significant generational heterogeneity only for the Innovativeness → Dairy Consumption path. No significant generational differences were found for the effects of Nutritional Value or Sustainability. Therefore, differences in the significance or magnitude of these coefficients across individual generational models should be interpreted as descriptive patterns rather than as evidence of statistically significant between-group differences. 

Generational cohorts develop their eating habits under different social, economic, and cultural conditions, which may influence their current preferences, criteria for evaluating products, and willingness to change established behaviors. Potentially relevant factors include the availability of milk and its alternatives during childhood and adolescence, dietary recommendations prevalent at the time, the economic situation of households, the development of the food market, and changing perceptions of health, animal welfare, and the environmental impact of food production [28,29].

Baby Boomers

The BB generation comprises people born during the two post-Second World War decades, when birth rates increased significantly in many regions, including Europe. Currently, BBs represent an important consumer group in the food market. Their dietary choices are shaped by their life experiences, a commitment to quality, and a growing concern for their health. Central and Eastern European Baby Boomers developed their dietary habits during a period marked by post-war food shortages, rationing, and limited product availability in many households [30]. These experiences may have strengthened their attachment to familiar, affordable, and nutritionally valued foods. For decades, BBs were key customers for dairy products. They grew up drinking milk or using it for the preparation of all meals, and enjoyed a variety of dairy products [31]. In this context, milk could have become established as a traditional component of the daily diet, with its consumption maintained through long-standing habits and family dietary patterns [32,33].

In our study, none of the three analyzed constructs was a statistically significant predictor of dairy consumption among Baby Boomers at the conventional significance level of *p* < 0.05. Therefore, the results do not provide sufficient evidence that any of these constructs predicted dairy consumption in this subsample.

Although the positive coefficient for innovativeness does not constitute evidence of a statistically significant relationship, its direction indicates that the present results do not support the interpretation that greater openness to innovative dairy products is associated with lower dairy consumption among Baby Boomers.

Previous research indicates that changing lifestyles, increasing consumer awareness, and consumer involvement may contribute to the development and acceptance of innovative food products [34]. Although Baby Boomers are frequently characterized as loyal consumers with strong brand preferences and established purchasing habits [31], this does not necessarily imply that they uniformly reject product innovation. Research conducted among individuals aged 60 years and older found that more than half of the respondents had never purchased functional dairy products, with particularly low purchase rates reported for lactose-free and cholesterol-lowering products. The authors also noted that older consumers tended to adopt a skeptical attitude towards this product category [35].

A stronger attachment to familiar products and established dietary patterns may help explain the continued presence of traditional dairy products in Baby Boomers’ diets. This does not, however, imply that older consumers reject all forms of innovation. Our research findings support the view that BBs are a relatively conservative group; however, they also highlight the heterogeneity within this generation. As values and beliefs regarding control influence priorities and behavior, the BB generation may represent a different group of older people compared to the stereotypical image of them—one that is more hedonistic, demonstrates greater independence, and is perhaps more demanding [36].

The absence of a significant effect of nutritional value on dairy product consumption does not necessarily indicate that nutritional attributes are unimportant to this generation. The literature confirms that health-related considerations are particularly important in the food choices of older adults, alongside taste, price, availability, and convenience of consumption [37].

The absence of a significant effect of sustainability may indicate that Baby Boomers’ food choices are more strongly associated with tradition, quality, and food origin. However, the BB generation exhibits behaviors characteristic of sustainable food consumption, including supporting local producers, choosing local and seasonal produce, and using leftovers. Members of this generation pay attention to organic certification, but their choices may be motivated more by health concerns than by a desire to reduce environmental impact [28].

Generation X

Generation X acts as a sort of buffer between the Baby Boomers and the Millennials. Gen X still adheres to some of the core values of the BB generation when it comes to grocery shopping, but also expresses opposition to factory farming and animal cruelty. Hence, the growing popularity of plant-based drinks and alternatives, which compete on the market with milk and dairy products. Generation X, like Baby Boomers, is aware of the high-fat content in dairy products [31]. Consumer research in Tennessee (US) showed that most BBs and Gen X purchased low-fat milk; BBs also purchased flavored milk the least, and yogurt was their most frequent purchase [38].

For Gen X, all constructs analyzed in the SEM, including nutritional value, sustainability, and openness to product innovations, proved to be significant predictors of dairy consumption. This pattern was observed within the Generation X subsample but should not, by itself, be interpreted as evidence that these effects were significantly stronger in this cohort than in the other generations. Formal cross-group tests did not demonstrate significant generational differences in the Nutritional Value → Dairy Consumption or Sustainability → Dairy Consumption paths. Significant overall generational heterogeneity was identified only for the Innovativeness → Dairy Consumption relationship.

The positive correlation between perceived nutritional value and dairy product consumption among Gen X may stem from the growing importance of preventive healthcare, physical fitness, and bone health at this stage of life [39]. Research also suggests that protein and physical activity are important for maintaining fitness and limiting the decline in functional ability as we age [40]. Members of Generation X may therefore view dairy products as a familiar and readily available source of nutrients. This interpretation is also supported by consumer research, in which health, nutritional value, and product familiarity were among the main factors influencing the choice of milk [41].However, because the formal cross-group test did not demonstrate significant generational differences in this path, this interpretation concerns the within-group association observed for Generation X and does not establish that nutritional value is more important for this cohort than for the other generations.

The positive link between a focus on sustainability and dairy consumption suggests that members of Generation X do not necessarily view this food category as incompatible with environmental responsibility. Previous research indicates that, when choosing dairy products, consumers take into account factors such as local origin, organic production methods, animal welfare, and environmental impact [42]. A focus on sustainable development may therefore involve not so much a complete rejection of dairy products as a more conscious choice of products that meet specific environmental and ethical criteria. Nevertheless, the formal cross-group test did not demonstrate significant generational differences in the Sustainability → Dairy Consumption relationship. This result should therefore be interpreted as a within-group association rather than evidence that sustainability plays a stronger or distinct role among members of Generation X.

Within the Generation X model, openness to innovation showed the numerically strongest positive association with dairy product consumption among the three constructs analyzed for this cohort. This may indicate that more innovative members of Generation X are receptive to new dairy products, including lactose-free, high-protein, fortified, functional, and convenience-oriented products. Product innovation may therefore support dairy consumption by adapting this product category to consumers’ changing health needs, lifestyles, and expectations. The result does not support the previous interpretation that more innovative members of Generation X are less attached to dairy products or more likely to replace them with plant-based alternatives [41]. In contrast to the results for nutritional value and sustainability, the formal omnibus comparison confirmed significant generational heterogeneity in the Innovativeness → Dairy Consumption path. However, without pairwise post-hoc comparisons, the analysis does not establish which specific generational groups differed significantly from one another.

Generation X can therefore be regarded as a group that combines a belief in the high nutritional value of dairy products with a sensitivity to sustainability issues and a willingness to embrace new market solutions.

Generation Y

Generation Y, also known as the Millennials, grew up during a period of rapid socio-economic development, the rise of the internet, mobile telephony, and globalization, all of which have had a significant impact on their values and lifestyle.

Among the surveyed Millennials, sustainability was the only significant predictor of dairy product consumption; additionally, this relationship was positive. The standardized coefficient for sustainability was numerically the largest among the generational models. However, the formal cross-group test did not demonstrate a statistically significant generational difference in the Sustainability → Dairy Consumption relationship. Therefore, this finding should be interpreted as a significant within-group association and a descriptive tendency rather than evidence that sustainability was more strongly associated with dairy consumption among Millennials than among the other generations. These findings are consistent with the characteristics of Millennials as consumers who are relatively sensitive to environmental and social issues, such as climate change, animal welfare, local sourcing, and responsible food production [43]. This generation is closely connected to the internet and social media. As a result, they have been particularly exposed to messages from groups opposed to dairy consumption that highlight the negative effects of consuming dairy products. These concerns have led Millennials to turn to plant-based alternatives. Millennials also want to feel an emotional connection with the companies from which they buy food—they pay attention to sustainability practices and how a company can help the planet [31]. Scozzafava et al. (2020), like Madududu et al. (2024), found that providing Millennials with information on environmental sustainability and animal welfare increased their willingness to pay for organic milk compared with cow’s milk [42,44].

Other studies have shown that a defining characteristic of Generation Y is their combination of environmental awareness with a pragmatic approach to food choices. Although Millennials express an interest in responsible production and reducing the negative environmental impact of consumption, research indicates that their purchasing decisions remain dependent on price, quality, taste, convenience, and product availability [45,46].

Results of research conducted in Poland in 2014 demonstrated that Millennials were not homogeneous in their environmental consciousness; however, they were generally aware of the various negative consequences of in-house food management practices. Despite this, during grocery shopping, the majority of respondents focused on determinants typically associated with food (taste, quality, healthy eating), rather than on environmental and social values. Millennials accept sustainable products and services when they are directly beneficial to them (fitness, health, economic value, convenience) and align with their preferences and expectations [47]. They are looking for food that combines environmental values with practical and sensory benefits. This generation interprets sustainability quite broadly, and it can encompass environmental protection, reducing food waste, animal welfare, social fairness, and supply chain transparency [43]. Furthermore, Aizaki and Takeshita [48] found in their research that Millennials attach varying levels of importance to many aspects of sustainable dairy production, including, among others, reducing greenhouse gas emissions, protecting biodiversity, animal welfare, minimizing waste, and supporting rural communities. Millennials also seem to be more likely to adopt flexible eating patterns, embracing the flexitarian model. Rather than completely eliminating animal-based products, they may reduce their consumption, alternate between traditional and plant-based options, or favor products perceived as more responsible [49,50,51].

Generation Z

Among consumers classified in our study as Generation Z, none of the three analyzed SEM constructs were statistically significant predictors of dairy product consumption. This finding suggests that Gen Zs—also known as iGen, Digital Natives, Post-Millennials, Plurals, and Founders—represent a specific type of consumer whose purchasing and eating habits have been shaped by new factors within their food environment. This group is considered the first true digital-native generation [52]; therefore, their digital fluency shapes how they engage with the world and adapt to rapid technological change, while balancing sustainable lifestyles amid global challenges [53,54]. Although many of them support or engage in environmental activism, research focuses mostly on their consumption patterns, highlighting values such as sustainability and minimalism [55,56,57,58].

Gen Zs were born during a period of dynamic social, economic, and cultural change and development in Central Europe. In Germany, the reunification process was underway (from 1990), while in Poland and the Czech Republic, preparations were being made for accession to the European Union in 2004. They grew up amidst rising prosperity and the development of the internet, IT, electronics, e-commerce, and online markets. Material prosperity went hand in hand with psychological and social development, but the formation of these bonds—particularly among Post-Millennials—was severely disrupted by the COVID-19 pandemic. As a result, Gen Zs expect exceptional convenience, authenticity, and strict alignment with their values. They did away with the traditional marketing funnel, as social media has become their marketplace. They simultaneously search for information, verify offers, and make purchases on these platforms. To win their loyalty, manufacturers must offer tangible quality, transparent pricing, and ethical practices [59].

Gen Zs view consumption as a reflection of their individual identity. They are attracted to niche products, limited drops, and brands that offer customization. They prefer to engage with companies that build with them rather than market at them, often participating in community-driven feedback loops [60].

Since this generation grew up during a period of rapid development and the increasing availability of new food categories, the range of plant-based beverages and lactose-free products expanded considerably over their lifetimes. These products became widely available as alternatives to conventional ones in many regions [61,62]. In Poland, although technology for developing lactose-free products has been in place for 30 years, these products only began appearing on the market in larger numbers in the mid-2010s. A recent study of Polish consumers’ knowledge and purchase behavior [63] showed that only 13% of young respondents reported problems digesting lactose, but only half of these individuals bought lactose-free dairy products. In another study among youth [64], only 3.4% of respondents stated they recognize and consume lactose-free products. Research on Generation Z confirms its familiarity with plant-based foods and alternative protein sources, as well as its relatively high willingness to incorporate them into the diet [65]. As a result, members of this generation operate within a more diverse food environment than previous generations.

Dairy products may be perceived by Gen Z consumers as one of many available food categories. Research has shown that the food choices of this generation are often shaped by taste, price, convenience, packaging, and label information [66], as well as by digital content, emerging trends, and recommendations shared on social media [67].

A recent comparative analysis of Gen Z individuals living in two European capital cities found that this generation, grappling with unprecedented challenges, is adopting a more comprehensive hybrid lifestyle, including shared housing, hybrid work, and environmentally conscious practices. The socio-spatial impacts of digitalization and the escalating global environmental crisis driven by climate change pose challenges that were less prominent or entirely absent for previous generations. Hybrid lifestyles emerge as adaptive responses to the challenges of the environments in which they live [68]. Although such lifestyles may also be related to the food environment and food choices, this mechanism was not measured in the present study and should therefore be regarded as a hypothesis for future research rather than a conclusion derived from our current findings.

Gender in shaping dairy product consumption

Additional gender-specific analysis showed that sustainability was a positive predictor of dairy product consumption among both women and men. Among women, nutritional value was an additional positive predictor, consistent with previous studies indicating that women attach greater importance to the health implications of their food choices and to the quality of their diet [69]. Innovativeness was not significantly associated with dairy consumption among women. Among men, openness to innovation was positively associated with dairy product consumption, whereas nutritional value was not a significant predictor. This may suggest that more innovative male consumers are more receptive to new or modified dairy products, such as lactose-free, high-protein, fortified, or functional products. This interpretation is consistent with previous research linking consumer innovativeness with greater openness to the adoption of novel food products [17]. However, formal cross-group comparisons did not show significant gender differences in the effects of nutritional value, Δχ^2^(1) = 0.18, *p* = 0.671; sustainability, Δχ^2^(1) = 0.29, *p* = 0.589; or innovativeness, Δχ^2^(1) = 2.98, *p* = 0.084, on dairy consumption. Therefore, the different significance patterns observed within the female and male subsamples should not be interpreted as evidence that gender moderates these relationships.

## 5. Strengths and Limitations

One of the major strengths of the study was the sample size, comprising 3131 consumers living in Poland, the Czech Republic, and Germany. Respondents were selected to ensure that national samples were representative, thereby enhancing the reliability of the results. A further advantage was the use of structural equation modeling, which enabled the simultaneous assessment of the importance of perceived nutritional value, sustainability orientation, and openness to innovativeness as predictors of dairy consumption. Furthermore, the multi-group analysis enabled us to formally evaluate whether the structural relationships varied across generations and gender groups. These comparisons supported generational heterogeneity only for the Innovativeness → Dairy Consumption path and did not demonstrate significant gender differences in any of the analyzed relationships.

The study findings also have valuable practical implications for the dairy sector. They can support the design of products and information campaigns tailored to the diverse needs, expectations, and motivations of different generations. Taking a generational perspective into account can help better align product characteristics, how their nutritional and environmental value is communicated, and the scope of innovations introduced with the specific characteristics of individual consumer groups.

However, the study also has certain limitations. Its cross-sectional nature does not allow conclusions to be drawn regarding cause-and-effect linkages. Given the generally small to moderate magnitude of the observed associations, the findings should be interpreted with caution and not regarded as evidence of causal relationships.

The interpretation of intergenerational differences also needs further study, as belonging to a particular generation is closely linked to age and one’s current stage of life. The observed correlations may stem from experiences characteristic of a given generation, their current health needs, family circumstances, income levels, and lifestyle, or from the differences in dairy consumption across countries; therefore, the potential impact of this variable should be taken into account in subsequent analyses. The data were self-reported and may therefore have been subject to recall bias and social desirability bias. Nevertheless, the generational approach remains a useful interpretative tool, enabling a better understanding of the diversity of behaviors related to dairy product consumption.

## 6. Conclusions

Dairy consumption among Central European adults was positively associated with perceived nutritional value, sustainability considerations, and consumer innovativeness, with sustainability showing the strongest association in the overall sample. Although the generation-specific models showed different within-group patterns, formal cross-group comparisons demonstrated significant generational heterogeneity only for the Innovativeness → Dairy Consumption path. No significant generational differences were identified for nutritional value or sustainability, and none of the examined structural relationships differed significantly by gender.

The study findings demonstrate the multidimensional nature of dairy consumption while indicating that generational and gender-specific patterns not supported by formal cross-group tests should be interpreted descriptively. Further research, particularly using longitudinal designs, is needed to confirm these relationships and assess their relevance to public health nutrition communication, policy-making, and dairy product development at both regional and country levels.

## Figures and Tables

**Table 1:** Characteristics of the study sample.

Variables	Total Sample N = 3131 (100%)	Polish N = 1508 (100%)	German N = 812 (100%)	Czech N = 811 (100%)	*p*-Value ^#^
Gender					
Women	1623 (51.84)	794 (52.65)	404 (49.75)	425 (52.40)	0.3833
Men	1508 (48.16)	714 (47.35)	408 (50.25)	386 (47.60)	
Generation (years, min.–max.)					
Baby Boomers (60–78)	531 (16.95)	214 (14.19)	178 (21.92)	139 (17.14)	<0.0001
Generation X (44–59)	974 (31.11)	463 (30.70))	261 (32.14)	250 (30.83)	
Generation Y (28–43)	1097 (35.04)	592 (39.26)	242 (29.81)	263 (32.42)	
Generation Z (18–27)	529 (16.90)	239 (15.85)	131 (16.13)	159 (19.61)	

# *p* value of test of independence chi^2^.

**Table 2:** Factor loadings and the assessment of internal consistency in a CFA model.

Construct	Variable/Item	Cronbach’s Alpha	Factor Loading	CR	AVE
Nutritional Value	Rich in vitamins and minerals	0.842	0.766	0.858	0.440
	Beneficial to health		0.785		
	Nutritious		0.737		
	High in protein		0.692		
	Tasty		0.566		
	Contain no artificial additives		0.708		
	Have no genetically modified ingredients		0.595		
Innovativeness	In general, I am among the last people in my circle of friends to purchase a new dairy product *(R)	0.637	0.705	0.681	0.334
	If I heard that a new dairy product was available in a local store, I would be interested in buying it		0.226		
	Compared to my friends, I am less likely to buy new dairy products *(R)		0.638		
	I would consider buying a new dairy product even if I had not heard of it before		0.371		
	In general, I am among the last people in my circle of friends to know the names or brands of new dairy products *(R)		0.616		
	I know more about new products than others do		0.476		
Sustainability	I choose dairy products that are produced in an environmentally friendly way	0.839	0.787	0.842	0.474
	I pay attention to whether dairy products are packaged in an environmentally friendly way		0.791		
	I consider the place of origin of dairy products to reduce transport distances and their carbon footprint		0.725		
	I choose dairy products with quality certifications		0.636		
	Environmental pollution affects my daily life		0.609		
	I am aware of the impact of food consumption on planetary health		0.546		

* (R)—reverse-coded item.

**Table 3:** Metric invariance testing parameters.

Grouping	Model	χ^2^	df	CFI	TLI	RMSEA	CMIN/DF	ΔCFI	ΔRMSEA
Generation	Configural	2803.9	609	0.956	0.947	0.020	4.604	−0.002	0.000
	Metric	2972.8	645	0.954	0.950	0.020	4.410		
Gender	Configural	2803.9	609	0.956	0.947	0.020	4.604	0.000	−0.001
	Metric	2823.9	619	0.956	0.948	0.019	4.562		

**Table 4:** Model results across generations.

Endogenous Value		Latent Value	Estimate	S.E.	C.R.	*p*	Standardized Estimate (β)
ALL							
Dairy Consumption	<---	Nutritional Value	0.018	0.006	2.72	0.007	0.089
Dairy Consumption	<---	Sustainability	0.064	0.010	6.31	<0.001	0.237
Dairy Consumption	<---	Innovativeness	0.025	0.009	2.69	0.007	0.092
Generation BB							
Dairy Consumption	<---	Nutritional Value	0.011	0.007	1.519	0.129	0.160
Dairy Consumption	<---	Sustainability	0.021	0.011	1.834	0.067	0.229
Dairy Consumption	<---	Innovativeness	0.027	0.015	1.914	0.056	0.255
Generation X							
Dairy Consumption	<---	Nutritional Value	0.032	0.010	3.254	0.001	0.189
Dairy Consumption	<---	Sustainability	0.036	0.013	2.837	0.005	0.161
Dairy Consumption	<---	Innovativeness	0.050	0.02	3.372	<0.001	0.209
Generation Y							
Dairy Consumption	<---	Nutritional Value	0.007	0.008	0.916	0.360	0.047
Dairy Consumption	<---	Sustainability	0.069	0.018	3.804	<0.001	0.344
Dairy Consumption	<---	Innovativeness	−0.014	0.011	−1.302	0.193	−0.072
Generation Z							
Dairy Consumption	<---	Nutritional Value	0.037	0.021	1.745	0.081	0.216
Dairy Consumption	<---	Sustainability	0.002	0.017	0.117	0.907	0.007
Dairy Consumption	<---	Innovativeness	0.003	0.016	0.193	0.847	0.011

**Table 5:** Model effects by gender.

			Estimate	S.E.	C.R.	*p*	Std. Estimate (β)
Female							
Dairy Consumption	<--	Nutritional Value	0.024	0.011	2.236	0.025	0.105
Dairy Consumption	<--	Sustainability	0.068	0.016	4.194	*p* < 0.001	0.215
Dairy Consumption	<--	Innovativeness	0.009	0.014	0.687	0.492	0.032
Male							
Dairy Consumption	<--	Nutritional Value	0.011	0.007	1.521	0.128	0.069
Dairy Consumption	<--	Sustainability	0.060	0.012	4.795		0.269
Dairy Consumption	<--	Innovativeness	0.036	0.012	2.950	0.003	0.150

*p* < 0.001. Multi-group analysis was conducted to examine whether the structural relationships differed across generational cohorts. Equality constraints were imposed separately on each structural path, and the constrained models were compared with the corresponding unconstrained models using chi-square difference tests.

**Table 6:** Model fit statistics.

CMIN		Baseline Comparisons		RMSEA	
NPAR	294	NFI (Delta1)	0.930	RMSEA	0.018
CMIN	3706.5	RFI (rho1)	0.917	LO 90	0.018
DF	903	IFI (Delta2)	0.946	HI 90	0.019
P	0.000	TLI (rho2)	0.936	PCLOSE	1.000
CMIN/DF	4.105	CFI	0.946		

## Data Availability

The data is the property of the Warsaw University of Life Sciences in Warsaw. Any further inquiries about presented data can be directed to the corresponding authors.
